# Hydrogen Recovery from Waste Aluminum–Plastic Composites Treated with Alkaline Solution

**DOI:** 10.3390/ma15238699

**Published:** 2022-12-06

**Authors:** Olesya A. Buryakovskaya, Mikhail S. Vlaskin

**Affiliations:** Laboratory of Energy Storage Substances, Joint Institute for High Temperatures of the Russian Academy of Sciences, 125412 Moscow, Russia

**Keywords:** aluminum–plastic composites, multilayer packages, alkali solution, hydrogen evolution

## Abstract

An alternative solution to the problem of aluminum–plastic multilayer waste utilization was suggested. The process can be used for hydrogen generation and layer separation. Three different sorts of aluminum–plastic sandwich materials were treated with an alkali solution. In the temperature range of 50–70 °C, for tablet blisters of polyvinylchloride and aluminum (14.8 wt.%), the latter thoroughly reacted in 15–30 min. For sheets of paper, polyethylene, and aluminum (20 wt.%), full hydrogen ‘recovery’ from reacted aluminum component took 3–8 min. From the lids of polyethylene terephthalate, aluminum (60 wt.%), and painted polyethylene with perforations, the aluminum was consumed after 45–105 min. The effect of perforations was the reduction of the process duration from nearly 90 min for the lids with no perforations to nearly 45 min for the perforated ones (at 70 °C). Perforations provided better contact between the aluminum foil, isolated between the plastic layers, and the alkali solution. Hydrogen bubbles originating near those perforations provided foil separation from the upper painted plastic layer by creating gas gaps between them. The remaining components of the composite multilayer materials were separated and ready for further recycling.

## 1. Introduction

The historical development of the global energy industry was largely supported by the transition to more concentrated and convenient fuels. Although fossil fuels still prevail over the renewables, in recent years, a clear course to reduce carbon dioxide emissions has been established. Within the coming years, a large share of the energy produced from hydrocarbons is expected to be taken by ‘green’ energy sources [1,2,3]. Hydrogen is considered the most promising energy carrier of the future. According to the forecasts, ‘blue’ and ‘green’ hydrogen will be generated in industrial amounts from fossils or water (by electrolysis) using solar, wind, geothermal, tidal, or hydro power [4,5,6,7,8,9,10,11,12]. An important method for hydrogen production, which is worthy of note, is solar-driven hydrogen production by photocatalytic water splitting in the presence of specially designed complex nanocomposites [13,14]. Gas turbines and fuel cells fueled with hydrogen can be widely used for the power (and heat) supply of buildings and urban or marine vehicles [15,16,17]. However, serious precautionary measures should be ensured to provide safe hydrogen storage, transportation, and utilization due to fire and explosion risks [18,19,20].

A safe and convenient method to provide the required amount of hydrogen in situ is the implementation of the reaction between water and hydro-reactive metals, such as magnesium and aluminum. Aluminum is protected against oxidation with a thin oxide film on its surface. Therefore, to induce its reaction with water, some activation measures should be applied. Most common ones include temperature elevation above 100 °C to accelerate the reaction between aluminum and liquid water or water vapor [21,22,23,24,25,26], aluminum modification with Ga-based alloys to promote its fracturing along grain boundaries [27,28,29], and the preparation of alloys or composite powders with different metals (e.g., Cu, Bi, In, Sn, Fe, Si, and Ni) [30,31,32,33,34,35,36,37,38,39,40] to enhance aluminum corrosion with hydrogen evolution. Aluminum ball milling together with oxides or hydroxides (Bi(OH)_3_, Al(OH)_3_, Al_2_O_3_, CaO, TiO_2_, etc.), carbon-based materials (graphene, graphite, and carbon nanotubes) and salts (such as NaCl, KCl, NiCl_2_, and CoCl_2_) is commonly used to reduce the particle sizes, destroy the oxide film, and create crystal lattice imperfections [41,42,43,44,45,46,47,48,49,50,51,52,53,54,55]. The surface oxide layer can also be destructed during external loading. Thus, for instance, solution treated, cold rolled, or aged samples were embrittled by hydrogen originating from the aluminum reaction with atmospheric water and trapped at different sites (interstitial lattices, dislocations, grain boundaries, S′-phase, and vacancies), while those that had been ultrasonic shot-peened avoided severe embrittlement [56,57]. Acid or alkali aqueous solutions are implemented in order to ‘chemically’ remove the protective film via the transformation of the aluminum oxide into soluble compounds (e.g., aluminates, oxy-, and hydroxychlorides) [58,59,60,61].

The implementation of alkali (mainly NaOH and KOH) was found to be a promising solution to ensuring the fast oxidation of aluminum in a bulk form, or waste aluminum materials (cans, foils, dross, machining chips, and powders) under relatively moderate temperatures (20–90 °C) [62,63,64,65,66,67,68]. A number of early studies tested aqueous alkali solutions with relatively high concentrations (1–10 M) [69,70,71,72,73,74,75,76]. However, it was established that lower alkali concentrations (0.1–1 M or less) can be effectively used [77,78,79,80,81,82,83,84]. In research [61], it was established that at 18 °C, NaOH reacted with Al powder slowly (<50% H_2_ yield for 40 min), while at 40 °C, 100% was achieved in less than 20 min. In an early study [85], it was shown that the amount of aluminum exceeding the stoichiometric value for its reaction with NaOH with NaAl(OH)_4_ formation was not converted into H_2_. However, studies [86] and [87], in which higher temperatures were employed, demonstrated the effect of alkali regeneration with the precipitation of Al(OH)_3_. Moreover, later research proved that under intensive mixing, a KOH solution with a low concentration (0.1 M) provided high hydrogen yields for aluminum chips and granules with rather a high aluminum-to-water mass ratio of 1:4, and in articles [88,89,90], it was established that under the same concentrations, NaOH provided a faster reaction than KOH.

Hydrogen generation from the oxidation of low-grade, secondary, or waste aluminum is obviously more profitable than its production by water splitting with a brand new metal. Besides used aluminum foils, wires, dross, cans, machining products, construction parts, debris, and other waste, there are a number of plastic-based composite sandwich materials that contain an aluminum layer. Those multilayer materials include packaging for medicines (e.g., tablet and capsule blisters, sachets for hot drink powders), spices, food (lids for yogurt or cottage cheese containers, packages for butter and curd cheese bars), beverages and milk (Tetra Pak^®^ products), insulated cables, and sandwich sheets for construction. Aluminum foil pressed to layers of paper (PA), plastic, or some other material is bonded to them by mechanical adhesion (interlocking), and it cannot be separated mechanically. For that reason, the recyclability of such sandwich materials is poor, thus representing a complex problem. The most common plastic components of multilayer packaging are polyethylene terephthalate (PET), polypropylene (PP), and polyethylene (PE). Recent advances in their separation include the development of reversible cross-linking adhesives (for PET and PE) [91], and the implementation of different organic solvents (acetic acid, acetone, dimethyl formamide, ethanol, ethyl acetate, ether, dichloromethane, tetrahydrofuran, hexane, xylene, and toluene) [92,93,94,95]. One of the proposed methods for paper or paperboard-containing multilayer materials, includes package disintegration, filtering the cellulose pulp suspension, aluminum and polyethylene delamination with formic acid, and the extraction of microcrystalline cellulose using sulfuric acid [96]. Other techniques include pyrolysis, low-temperature torrefaction, hydrothermal liquefaction (HTL), and hydrothermal carbonization (HTC) [97,98,99,100]. For aluminum recovery, a number of methods have been proposed as well. Thus, it can be obtained from waste aluminum alloy by low-temperature molten salt electrolysis [101], acid leaching from pyrolysis products of waste printed circuit boards using sulfuric acid and hydrogen peroxide [102], recycling of carton packaging (75% paper, 20% polyethylene, and 5% aluminum) by pyrolysis at temperatures below 600 °C [103], aluminum recovery from waste composite laminates by sub- and super-critical water [104], and conventional processes of aluminum extraction in a rotary salt furnace of plasma furnace, scrap pretreatment by mechanical, pyrometallurgical, or hydrometallurgical techniques, smelting, and refining in reverberatory furnaces [105].

The main disadvantage of pyrolysis, torrefaction, HTL, and HTC is that those techniques do not provide the conversion of aluminum into hydrogen without its contamination with gaseous decomposition products (CH_4_, CO, and CO_2_) [98]. The major drawbacks of other separation methods include either their complexity, the long duration of treatment with solvents under moderate temperatures with low output [93], or the need for high temperatures and pressures [95]. Although some high-income countries launched pilot projects on the chemical separation of multilayer plastic packaging waste, due to their low cost efficiency, most waste of this type is either landfilled or incinerated. So, no mainstream solution for its recycling is forecasted to appear in the next 5–10 years [106].

For the above reasons, in the present study, an alternative approach to the utilization of multilayer materials is to be tested. The multilayer materials’ samples—tablet blisters of polyvinyl chloride (PVC) and aluminum foil (Al), PET–Al–PE lids for children’s curd containers, and PA–PE–Al sheets for curd cheese bars—will be treated with alkali solution to ‘dissolve’ the aluminum foil with hydrogen generation and separation of the residual layers. As for the selection of the alkali concentration and temperature range, the following plan was implemented. From the analysis of the abovementioned results on aluminum–alkali reactions, NaOH, due to its higher reaction rates, was preferred over KOH. From the consideration of the reported temperature dependencies, elevated temperatures (above 40 °C) were selected. Prior to conducting the experiments at the experimental facility, some ‘trials’ using a simplified system (magnetic stirrer with heating and glass Erlenmeyer flask) were carried out for 1 and 0.1 M NaOH at ~60 °C (without data recording). For the first concentration, quite a violent reaction was observed, while for the second, the process dynamics looked unimpressive. Although the synergetic effect of low-content KOH solution and intensive mechanical mixing for coarse aluminum was kept in mind, the accumulated amount of suitable original packaging materials was not large enough to profit from ‘mechanical frictional’ activation in the relatively big experimental facility’s reactor (1 L). Therefore, it was decided that an ‘intermediate’ NaOH concentration would be used of 0.5 M (the same for all experiments), a relatively moderate temperature range of 50–70 °C, and a mixing speed of 250 rpm. The proposed technique has the potential to be transformed into an effective solution for the important problem of aluminum–plastic waste utilization. Compared to the existing methods, the novel approach has the following prospective benefits: the use of a relatively low concentrated (up to 0.5 M) alkali solution and moderate temperatures (from 40 to 100 °C or lower), separation of the layers by the aluminum oxidation with hydrogen generation, and the absence of evident sources for severe hydrogen contamination (however, this should be specially tested).

## 2. Materials and Methods

The starting aluminum-containing materials used in each experiment included the following items. A double-layer composite with ‘uncovered’ foil surface was PVH–Al blisters for the following tablets: Nurofen^®^ (200 mg, Reckitt Benckiser Healthcare International Ltd., Nottingham, UK)—eight pieces; paracetamol (500 mg, JSC Pharmstandard-Leksredstva, Kursk, Russia)—four pieces; activated charcoal (250 mg, JSC Pharmstandard-Leksredstva, Kursk, Russia)—two pieces; and Carsil^®^ (35 mg, Sopharma JSC, Sofia, Bulgaria)—six pieces. A three-layer material of PA–PE–Al type was represented by a sheet for a curd cheese bar ‘Svitlogorie’ (50 g, JSC Dmitrovskiy molochnyi zavod, Dmitrovsk, Russia) cut into nine nearly equal pieces each. In addition, a multilayer PET–Al–PE composite sample was represented with two lids for children’s curd containers: ‘Agusha’ (100 g, JSC Wimm-Bill-Dann, Moscow, Russia) and ‘VkusVill’ (50 g, JSC Bryansky Gormolzavod, Bryansk, Russia) cut into four pieces of nearly the same size. An alkali aqueous solution was prepared using deionized water and analytical reagent grade NaOH pellets (Lachema Ltd., Praha, Czech Republic).

The experimental procedure included pouring 1000 mL of 0.5 M solution into a reactor (1000 mL, JSC Lenz Laborglas, Wertheim, Germany) and heating it with a heater (CC-308B; JSC ONE Peter Huber Kältemaschinenbau, Offenburg, Germany) under stirring with a magnetic mixer (C-MAG HS 7; JSC IKA-Werke, Staufen, Germany). A sample was then loaded into the reactor. The originating hydrogen passed through a Drexel flask into a glass vessel with water. The hydrogen volume was measured by a water ejection (water displacement) method representing a reliable widely used technique [82,107,108,109,110,111]. Water was ejected by the incoming gas to be collected in a flask and placed onto scales (ATL-8200d1-I; Acculab Sartorius Group, New York, NY, USA), whose readings were continuously transmitted to a computer. The temperatures in the reactor and glass vessel were measured, respectively, with an L-type thermocouple (TP.KhK(L)-K11; Relsib LLC, Novosibirsk, Russia) and a Pt100-type resistance temperature detector (TS-1288 F/11; Elemer LLC, Podolsk, Russia) connected to a multichannel thermometer (TM 5103; Elemer LLC, Podolsk, Russia). The atmospheric pressure was detected by a barometer (BTKSN-18; Technical Specification No. 1-099-20-85, UTYOS JSC, Ulyanovsk, Russia). The registered data were used to calculate the hydrogen volume values under standard conditions (Standard DIN 1343: 101,325 Pa, 0 °C) using the ideal gas law. For each sample and temperature point, three experiments were carried out.

The original multilayer samples, unreacted materials, and solid reaction product were investigated via X-ray diffraction (XRD) analysis performed using a Difraey 401 diffractometer (Scientific Instruments JSC, Saint Petersburg, Russia) with Cr-Kα radiation (0.22909 nm). The XRD patterns were processed using a database (Powder Diffraction File™) from the International Centre for Diffraction Data (ICDD). Visual investigation was carried out under a darkfield illumination by an optical microscope (Bio 6) equipped with a high-resolution camera (UCMOS 10000KPA; Altami LLC, Saint Petersburg, Russia). The aluminum foil thicknesses were assessed using Altami Studio 3.5 software and the calibration data. The general views of the original samples and remaining materials were captured by means of a Nikon D5200 camera with an objective AF-S DX Micro NIKKOR 40mm f/2.8G (JSC Nikon Europe, Amstelveen, The Netherlands).

## 3. Results

### 3.1. Blisters

**Figure 1:**
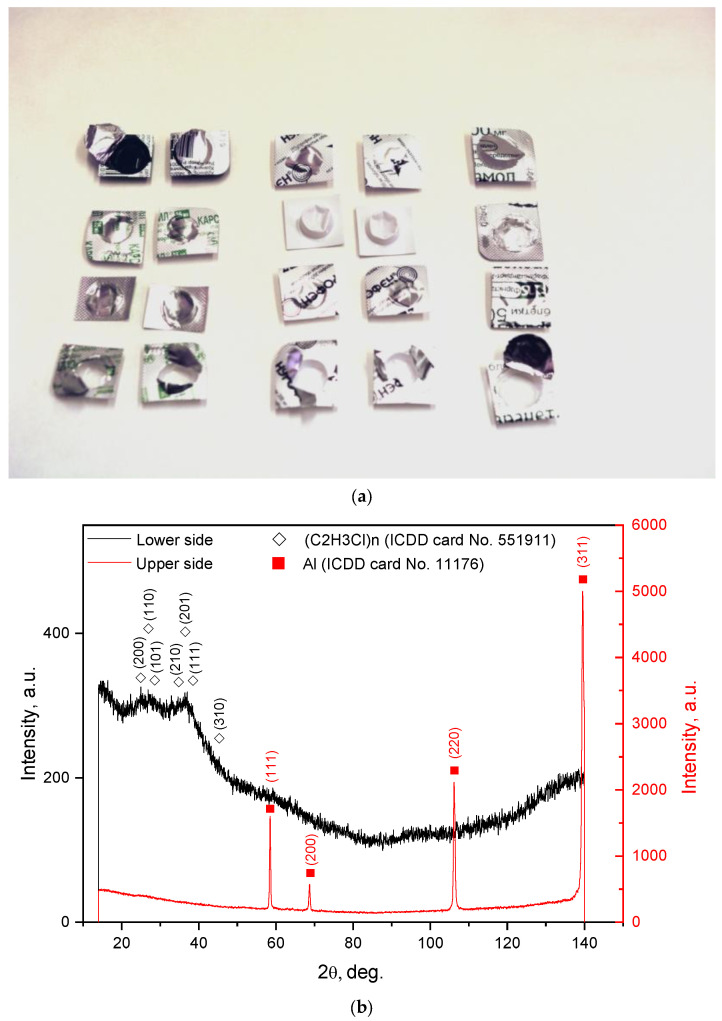
Characterization of a plastic–aluminum sample: (**a**) general view; (**b**) XRD patterns recorded from the lower (plastic) and upper (aluminum foil) sides.

The general view of the original plastic–aluminum sample (ensemble of tablet blister pieces) and its XRD patterns are given in Figure 1. The thickness of the aluminum layer evaluated by means of the optical microscope and camera was approximately 18–21 μm. The XRD pattern registered from the upper side corresponded to the face-centered cubic Al structure, and that recorded from the lower side corresponded to an amorphous polymer. The most common typical components of tablet blisters are PVC and aluminum foil. The first can be coated with PE, polyvinylidene dichloride (PVDC), or polychlorotrifluoroethylene (PCTFE), and the latter can be attached to an oriented polyamide (OPA) layer [112,113]. Earlier studies [114,115] revealed that PVC’s X-ray diffraction intensity was very low, so there were no sharp peaks in its XRD pattern. Although broad intensity peaks were obtained, they still matched with the PVC characteristics. The classic test for identifying PVC is to put it into a flame and observe the flame color and generated odor. A greenish-edged flame and smell of hydrochloric acid point to this material [116]. The said procedure was performed for the blisters: in the flame, a greenish section appeared, and the smoke had a typical smell. So, the main components of the blisters were PVC and Al.

**Figure 2:**
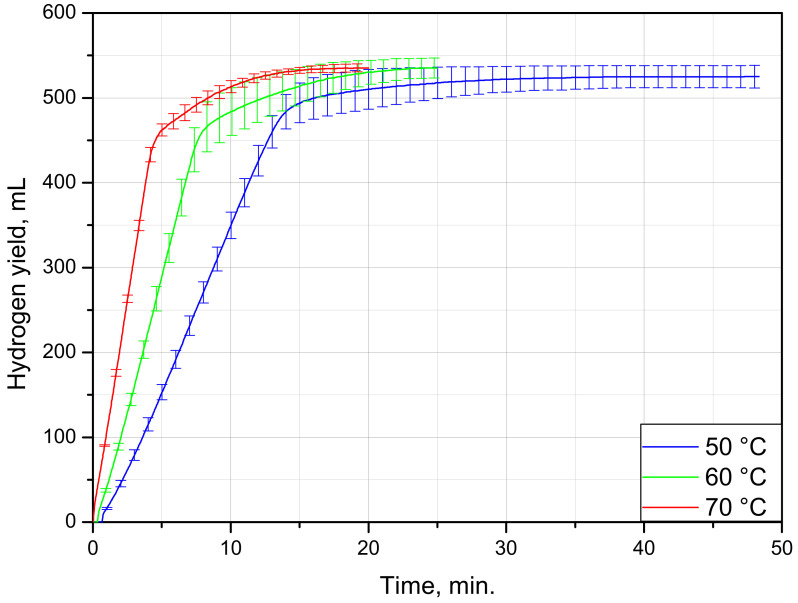
Kinetic curves for PVC–Al blisters under different temperatures.

The hydrogen evolution curves for the blister samples tested at different temperatures are shown in Figure 2. As can be seen from the plot, nucleation rapidly spread over the aluminum foil surface, so the initial acceleration stage typical for S-shaped topochemical was indistinguishable. All three curves were represented with the large section corresponding to the maximum reaction rate and adjacent ‘deceleration tail’. In the experiments at 50, 60, and 70 °C, the fastest reaction phase took correspondingly about 15, 7.5, and 5 min.

All of the samples had small differences in their total masses and foil surface areas. Therefore, their final hydrogen yields were close to each other. The data on the sample masses, foil areas, hydrogen yields, and maximum evolution rate are given in Table 1. On average, as much as 532 ± 10 mL of H_2_ was generated per a sample. Providing that the total amount of H_2_ per 1 g of pure Al is 1244 mL, and that its content in the food foil is ~98.3 wt.% (8011 and 8021 grades), the calculated average aluminum amount in a sample was 0.435 g. Dividing this value by the sample mass (2.945 g) gives 14.8 wt.% aluminum, which potentially can be converted into hydrogen.

**Figure 3:**
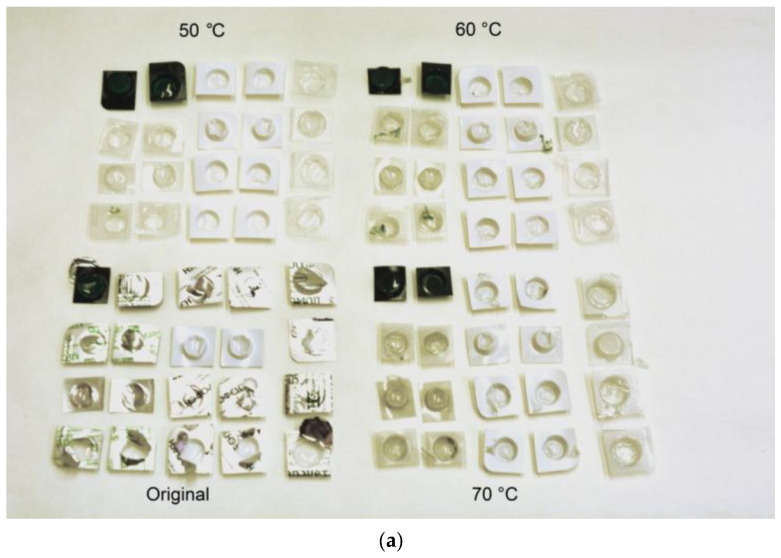

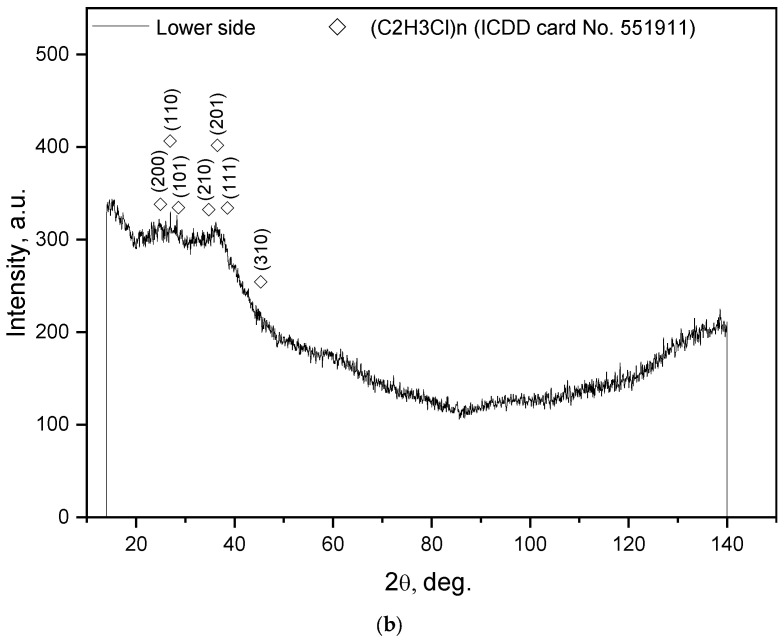
Characterization of the remaining sample components: (**a**) general view (original blisters and remaining components); (**b**) XRD pattern for the remaining components.

As can be seen in Figure 3, the remaining parts represent PVC blister pieces with no aluminum foil on them. The XRD pattern was similar to that of the PVC component of the original samples. A closer visual inspection of the remaining PVC pieces showed their shrinking after experiments at 70 °C. PVC is known to undergo dehydrochlorination in aqueous sodium hydroxide solution. This process was investigated in studies [117,118]; however, the experiments were carried out either under high temperatures (above 100 °C), or at high NaOH concentrations (20 wt.% and higher). Unfortunately, the measurements of the change in the PVC pieces’ mass and the analysis of hydrogen purity did not fall within the scope of the present ‘trial’ study. The solution composition and experimental conditions were selected such as to demonstrate the principle for aluminum foil removal from PVC blisters with hydrogen generation. To employ this principle in practice, further special investigations on adjusting the process parameters should be performed. Furthermore, the use of NaOH for sustaining the aluminum reaction with water is not obligatory, and some other solutions can be tested instead.

### 3.2. Sheets

**Figure 4:**
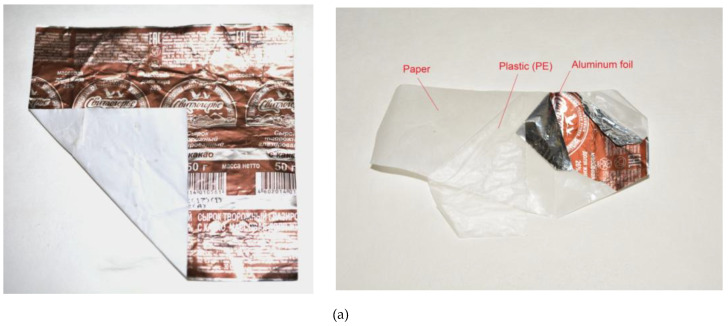

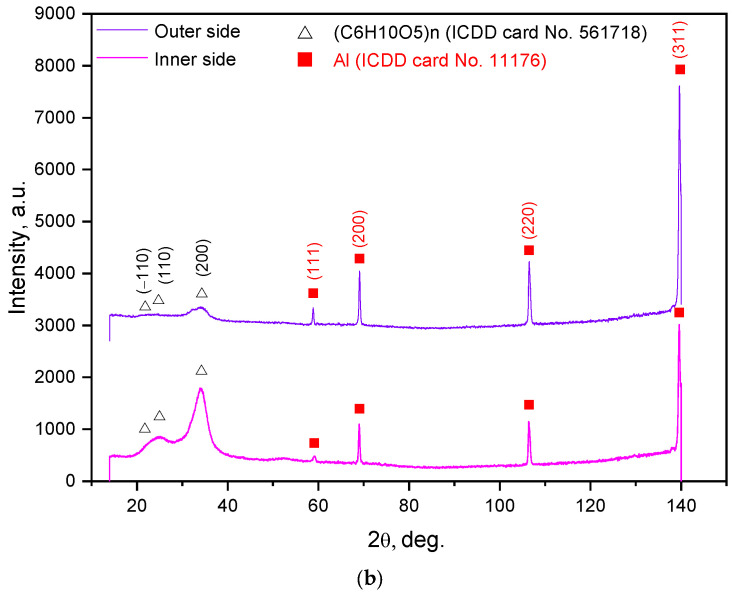
Characterization of paper–plastic–aluminum samples: (**a**) general view (original and with partially separated layers); (**b**) XRD patterns of the sample recorded from the inner (paper) and outer (aluminum foil) sides.

The general view of a tested paper–plastic–aluminum sample (original and with partially separated layers) and the respective XRD data are given in Figure 4. The sheets had an almost square shape (11.0 × 11.2 cm), and the aluminum layer thickness was approximately 6–8 μm. As can be seen, aluminum foil with painting applied onto it was the outer layer. The XRD patterns were obtained by scanning the sample’s inner side (paper layer) and outer side (aluminum foil with painting). Therefore, the intensities of the detected cellulose and Al phases differed in the two patterns. The XRD data did not prove the presence of the plastic interlayer separating the paper and aluminum layers that was apparently associated with its negligible thickness.

**Figure 5:**
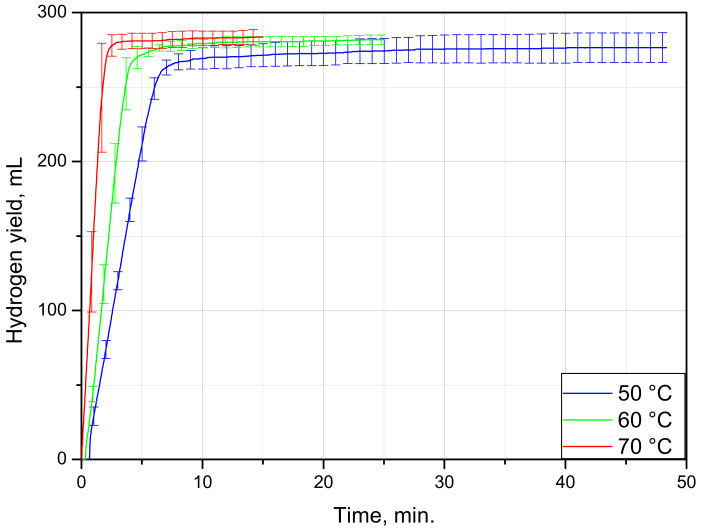
Kinetic curves for PA–PE–Al sheets under different temperatures.

The results of the experiments with the multilayer sheets are represented in Figure 5 and tabulated in Table 2. As can be seen from the plot, at the start of the reaction, all of the kinetic curves demonstrate a sharp rise from zero to several mL. This section is a result of the ‘inertia effect’ of the measuring system. To start pushing water out of the vessel, the accumulation of some amount of hydrogen was needed, and after starting the motion, the respective water volume was rapidly released. The major section of the kinetic curves corresponded to the maximum reaction rate with a short deceleration time. As can be seen, throughout almost the entire reaction process, the hydrogen was generated at an approximately constant rate. The entire aluminum surface of the samples was rapidly engaged in the process, and changes to its size was negligible until the very end. At 50, 60, and 70 °C, it took the aluminum in the samples nearly 8, 6, and 3 min, respectively, to be almost entirely consumed by the reaction. Such a fast process was apparently associated with an easy and fast detachment of painting from the foil surface.

The major reason for the divergences in the experimental data was related to accidental events during the experiments (e.g., possible stacking or overlapping of sample pieces and their rising to the surface in a random order), and a minor reason was associated with the possible difference in the structure of the tested samples (thicknesses of layers, distribution of deformations, their bending, etc.). The total hydrogen yields for all of the samples had close values: on average, they generated 281 ± 7 mL. Dividing this value by the total amount of hydrogen per 1 g of Al (1244 mL) and per its content in the food foil (~98.3 wt.% for 8011 and 8021 grades) gives the average aluminum content of ~0.23 g. Thus, the investigated packaging (1.139 g) contained ~20 wt.% of aluminum per piece, which can be converted into hydrogen.

**Figure 6:**
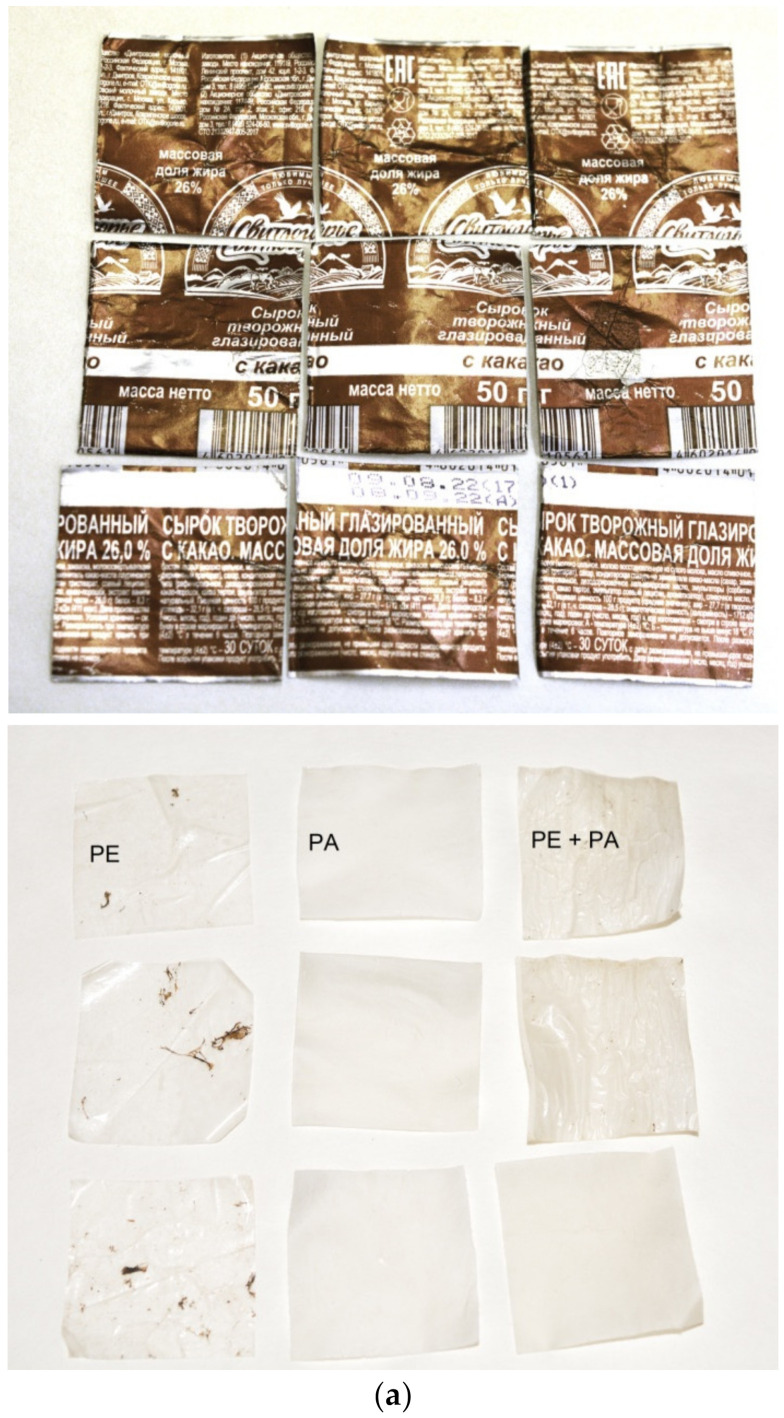

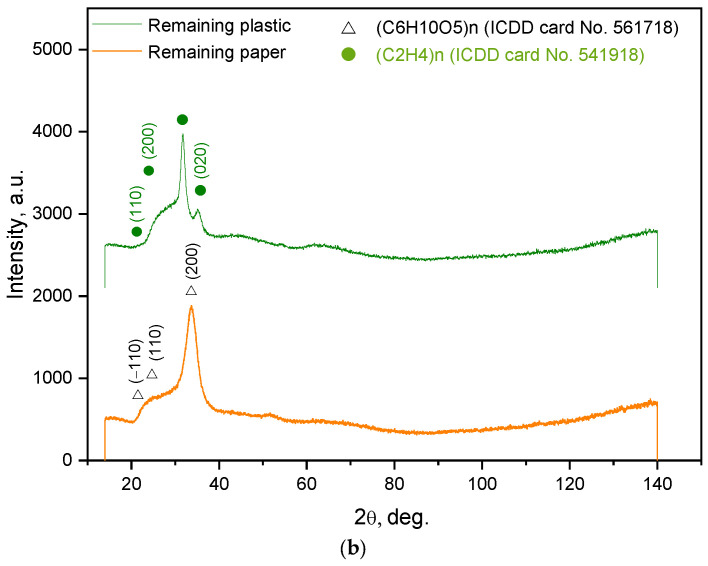
Characterization of the remaining sample components: (**a**) general view (original and residual sheet pieces); (**b**) XRD patterns for the remaining layers.

The general view of the remaining packaging components (and original for comparison) together with the corresponding XRD patterns are shown in Figure 6. For XRD scanning, several residual plastic pieces were placed on top of each other. The detected components were cellulose (PA) and polyethylene (PE). After longer durations of the experiments (at 50 °C), all nine sample pieces were separated into PA and PE layers, while after shorter time intervals, several pieces still had a loose connection between them. Some of the PE pieces had painting residuals on their surfaces, which were detached from the aluminum and stuck to them. PE is known to be stable in alkali solutions [119], and cellulose dissolves in NaOH solution [120]. Therefore, these materials are not expected to generate hydrogen contaminants. However, within the present study, no data on the interaction between the painting components and NaOH were obtained. Therefore, further studies are required to clarify the composition of the hydrogen ‘recovered’ from such PA–PE–Al sheets.

### 3.3. Lids

**Figure 7:**
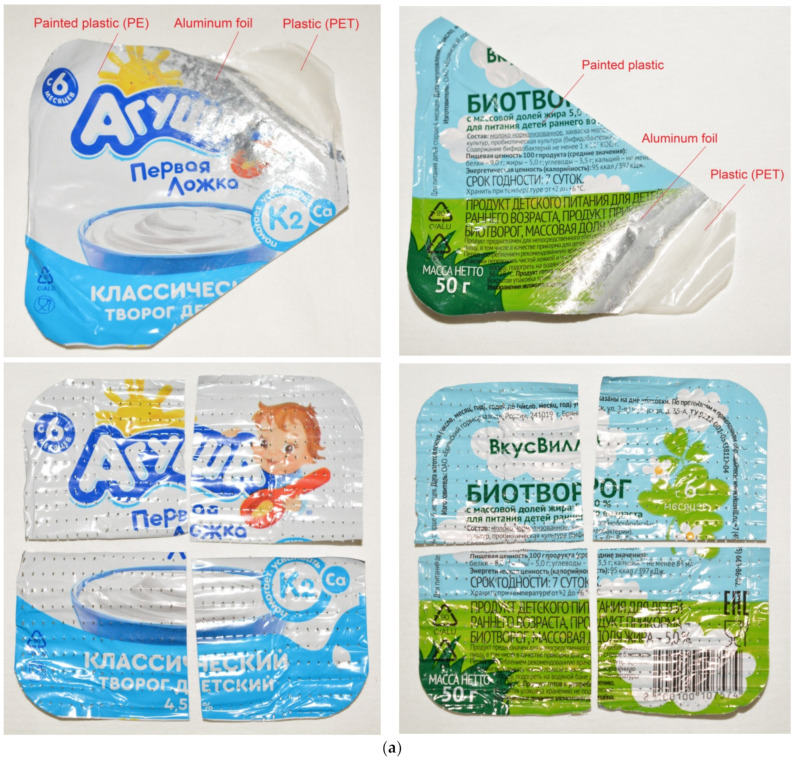

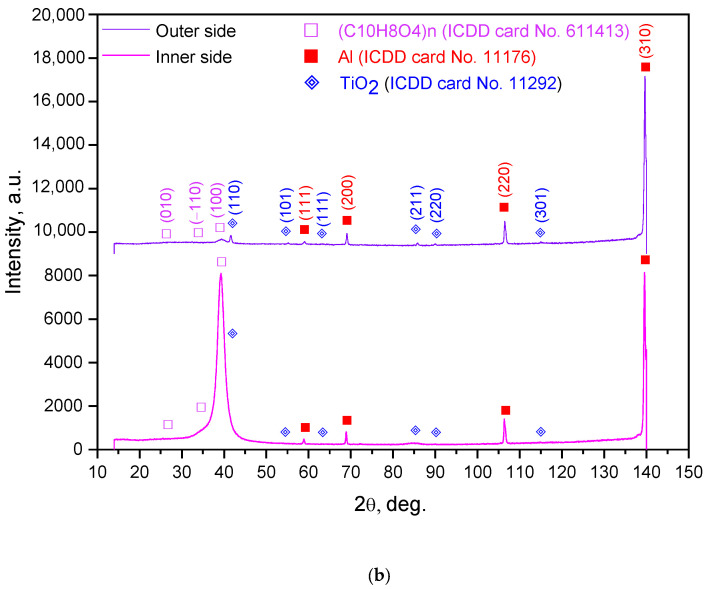
Characterization of plastic–aluminum–plastic samples: (**a**) general view (original and with partially separated layers); (**b**) XRD patterns of the sample recorded from the inner (plastic) and outer (painted plastic) sides.

The general view of the plastic–aluminum–plastic lids (with depicted underlayers and prepared for experiments) and XRD patterns are illustrated in Figure 7. In each experiment, a set of two lids (square 6.8 × 6.8 cm) composed of the same materials, but differing in their aluminum layer thickness (24–27 and 19–22 μm), were used. The perforation of the samples was achieved using a roller with 3 mm needles of 2.25 mm in diameter. The average number of perforations per lid was nearly 640 ± 30 pieces. XDR analysis clearly registered three phases: aluminum (Al), polyethylene terephthalate (PET), and titanium dioxide (TiO_2_). The sample was scanned from its inner (plastic) and outer (plastic with painting) sides, therefore the intensities of the components differed. The plastic layer with painting was not detected, apparently due to its low thickness. TiO_2_ represents a commercial white pigment for paint [121], and the plastic to which it was applied is highly likely to be polyethylene (PE), known to be a standard component of PET–Al–PE laminates widely used for dairy packaging [122,123,124,125].

**Figure 8:**
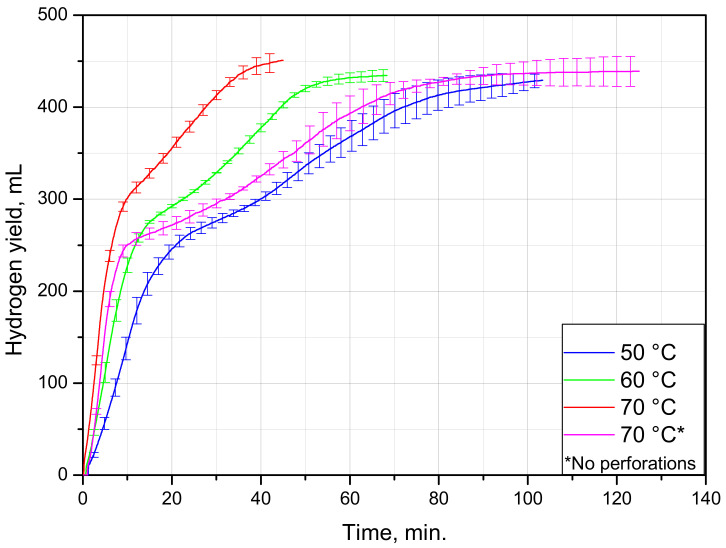
Kinetic curves for PET–Al–PE sheets under different temperatures.

**Figure 9:**
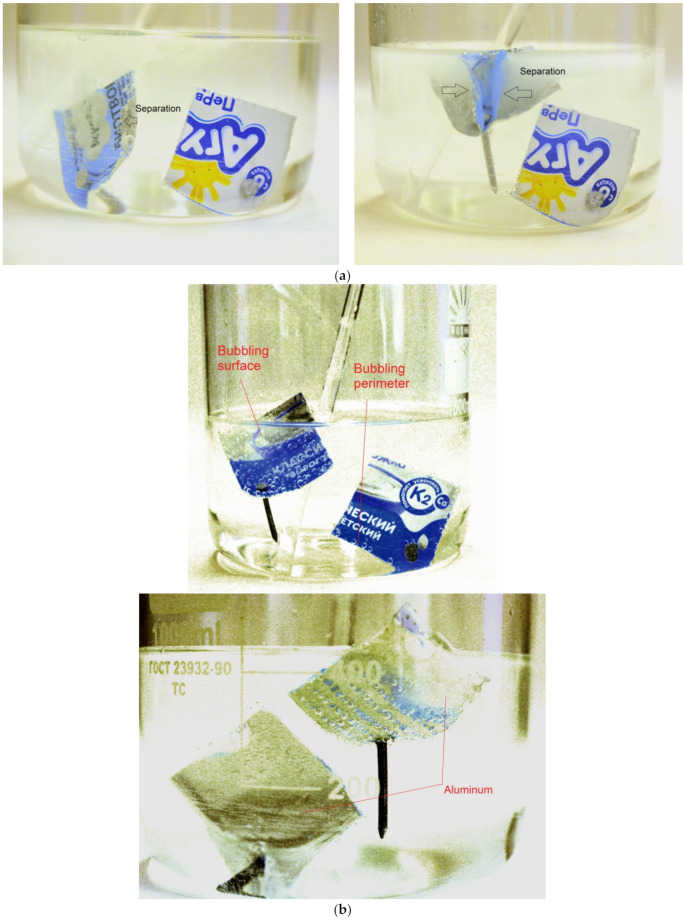
Illustration of the differences between different lid pieces: (**a**) difference between lids from different manufacturers; (**b**) difference between lids with and without perforations, front and rear views.

The kinetic curves and experimental data for PET–Al–PE lids are represented correspondingly in Figure 8 and Table 3. As can be seen from the plot, the curves have a steep part with the fastest reaction rate followed by another section corresponding to a slower reaction proceeding with further deceleration. Such an unusual shape of the curves was attributed to the fact that one of the lids tested in each experiment obviously had a thinner and looser upper plastic layer with painting. Soon after the experiment began, this layer readily peeled off from the foil and tore into tiny pieces. Upon uncovering the foil, the aluminum from these lids was soon consumed by the reaction. As the lids of the said type were heavier than those of another type, their contribution to the hydrogen yield was larger. The lids of the lighter type were ‘sealed’ with the painted layer much better. That was the reason why they had to undergo a longer treatment with friction during mixing and hydrogen bubbles, originating between the foil and outer PE layer and gradually detaching them from one another by the creation of gas gaps. As the lids of one type were ‘uncovered’ much faster than those of another, the maximum hydrogen evolution rates for the lids with and without perforations were almost the same. However, at the following reaction stage (when the ‘slower’ lids reacted), the effect of the perforations became obvious. Thus, at 70 °C, the perforated sample thoroughly reacted after nearly 45 min, while for the lids without perforations, it took about 90 min. The mentioned difference between lids from different manufacturers and the effect of perforations are illustrated by Figure 9.

In total, the PET–Al–PE lids produced 439 ± 12 mL of H_2_ per an average sample (two lids) of 0.594 g. From the calculations similar to those discussed in the previous subsections, it was found that the average aluminum content in the lids was as much as 60 wt.%. So, the samples of this type appeared to be very rich with aluminum.

**Figure 10:**
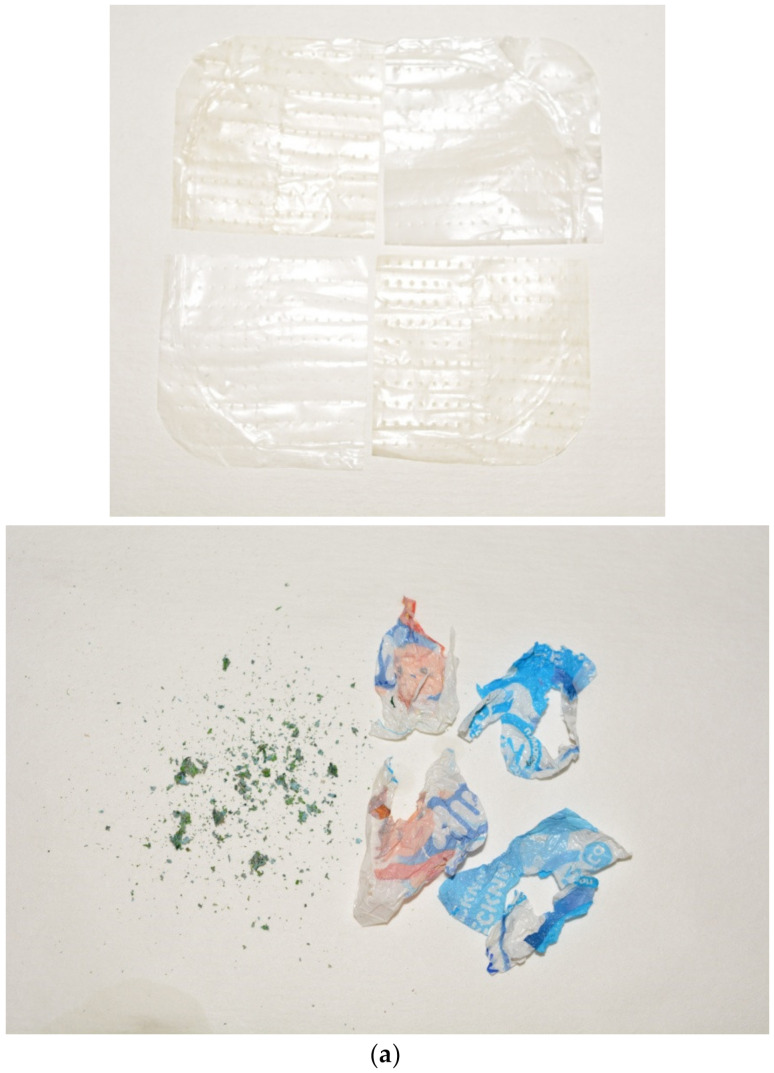

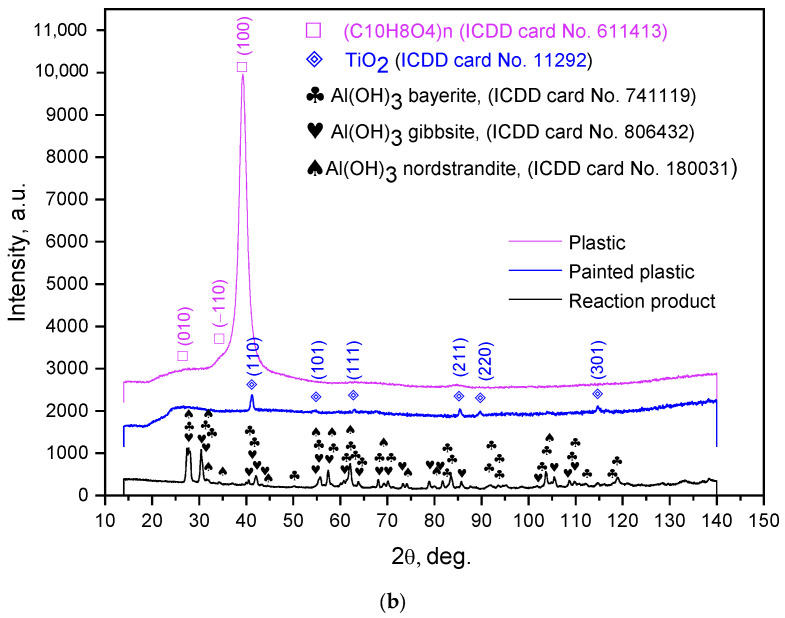
Characterization of the remaining sample components: (**a**) general view (residual plastic pieces: PET—left; painted PE—right); (**b**) XRD patterns for the remaining components.

The general view of the remaining plastic components (PET and painted PE) and the XRD patterns of them and the solid product of aluminum oxidation are shown in Figure 10. As can be seen, the PET layers did not undergo any visible drastic transformations compared with the original samples, while the layer of PE painted with TiO_2_ compound was torn into tiny pieces. The pieces of the denser painted layer were generally larger than those of the layer peeled off at the beginning. For the solid reaction product, three phases were identified. According to the XRD results, the aluminum transformed into three modifications of Al(OH)_3_: gibbsite, bayerite, and nordstrandite.

The PET from the tested multilayer material can undergo accelerated degradation in the presence of ethanol with NaOH acting as a catalyst [95,126]. Alongside this, 1 N NaOH solution was reported to cause PET degradation alone, in the temperature range of 60–70 °C [127]. As mentioned previously, PE is stable to alkali. However, in the present study, it was not established how painting was affected by NaOH and whether any hydrogen contamination takes place. This matter should be investigated in the course of another study.

## 4. Conclusions

The present study actually represented a trial study on hydrogen production from the oxidation of the aluminum component of multilayer materials, containing plastics and paper, in an alkali solution. It was established that the samples of PVC–Al and PA–PE–Al types, containing respectively 14.8 and 20.0 wt.% aluminum, demonstrated a fast start at the beginning of the process and rather high reaction rates. The samples of PET–Al–PE lids were consumed in a non-uniform manner: one of each couple of tested lids had a loose painted layer, which peeled off soon after the beginning, while the detachment of the denser painted layer from another lid took much more time. For the lids, the effect of perforation on the process speed was studied. It was found that for the lids with the denser PE layer, perforations provided faster foil separation from the upper layer and, therefore, its better contact with the alkali solution. Another potential advantage of perforation was that, compared to shredding, it allowed the dense pieces to remain almost unchanged in their shapes, while the less dense components reduced in size considerably. This could be convenient for separating large pieces from small-sized scrapings. The lids contained, on average, 60 wt.% of aluminum, which was successfully transformed into hydrogen.

The study demonstrated that hydrogen can be effectively recovered from plastic–aluminum and paper–plastic–aluminum sandwich materials. However, the purity of this hydrogen remains a matter for future studies. The result was the separation of the major components of the tested multilayer materials. The PA, PVC, and PET gravitated to the bottom, while PE floated to the surface. Moreover, the remaining materials can be separated by size, as some layers were torn into small pieces, while others retained their original sizes. For all of the residuals, large components (paper, PET, and PE sheets, PVC blisters) could be separated, for instance, by a large mesh net (e.g., woven media for liquid filtration) [128]. The smaller organic residuals (small plastic or painting rags) can be removed either by means of a small mesh filter (as the rags were still considerably larger than ultrafine Al(OH)_3_ particles) or separated by density using an appropriate salt solution, e.g., sodium poly-tungstate (1.4 g/mL), zinc chloride (1.5–1.7 g/mL), or sodium iodite (1.8 g/mL) [129]. In theory, Al_2_O_3_ (produced by aluminum hydroxide calcination) can be collected and used for Al production. In such a case, the mining and leaching stages of the process could be skipped. However, the costs for such utilization (including collection, storage, and transportation) should be calculated for each particular case. Recent advances in aluminum production included the creation of inert as opposed to sacrificial graphite electrodes, allowing manufacturers to perform a ‘carbon-free aluminum smelting process’ [130,131]. Moreover, the market price for Al is not constant, but undergoes cycles of growth and decline. So, the progress is non-stop, and aluminum can become more ‘eco-friendly’ and available. Summarizing all of the above, the process employed in the present study may have the potential for further improvement and be combined with the procedures for recycling the residual components.

## Figures and Tables

**Table 1:** Blister sample masses, foil surface areas, hydrogen yields, and maximum evolution rates.

Sample Mass, g	Foil Surface Area, cm^2^	Temperature, °C	H_2_ Yield, mL	Max. H_2_ Evolution Rate, mL/g/min
2.9907	77.94	50	540	98
2.8821	74.69	50	519	94
2.9124	75.53	50	516	99
3.0086	77.61	60	546	163
2.9832	76.91	60	536	152
2.9203	75.59	60	523	151
2.9349	75.77	70	537	250
2.9021	75.34	70	530	247
2.9684	75.87	70	539	248
**Average: 2.945 ± 0.044**	**Average: 76.14 ± 1.10**		**Average:** **532 ± 10**	

**Table 2:** Sheet masses, hydrogen yields, and maximum evolution rates.

Sample Mass, g	Temperature, °C	Hydrogen Yield, mL	Maximum H_2_ Evolution Rate, mL/g/min (Average)
1.1911	50	286	263
1.1125	50	277	229
1.0696	50	266	275
1.1129	60	278	409
1.1422	60	283	366
1.1501	60	284	320
1.1347	70	280	640
1.1406	70	281	582
1.1948	70	290	801
**Average: 1.139 ± 0.039**		**Average: 281 ± 7**	

**Table 3:** Lid masses, hydrogen yields, and maximum evolution rates.

Sample Mass, g	Temperature, °C	Hydrogen Yield, mL	Maximum H_2_ Evolution Rate, mL/g/min. (Average)
0.5971	50	432	65
0.6051	50	434	54
0.5882	50	422	53
0.5904	60	434	91
0.5942	60	428	81
0.6021	60	442	86
0.5901	70	453	130
0.5940	70	460	137
0.6025	70	440	150
0.5903 *	70	458 *	133 *
0.5893 *	70	432 *	130 *
0.5814 *	70	427 *	138 *
**Average: 0.594 ± 0.007**		**Average: 439 ± 12**	

* Lids without perforations.

## Data Availability

Not applicable.
